# Effects of Acute Time-Restricted Eating on Inflammation in Individuals With Psoriasis: Protocol for a Case-Control, Prospective Study

**DOI:** 10.2196/74999

**Published:** 2025-08-15

**Authors:** Sinibaldo Romero Arocha, Kim Han, Rebecca D Huffstutler, Geethika P Thota, Natalie A Macheret, Amber B Courville, Robert J Brychta, Kong Y Chen, Stephanie T Chung, Laura C Coates, Alexander J Clarke, Michael N Sack

**Affiliations:** 1 Kennedy Institute of Rheumatology University of Oxford Oxford United Kingdom; 2 Laboratory of Mitochondrial Biology and Metabolism National Heart, Lung, and Blood Institute Division of Intramural Research Bethesda, MD United States; 3 Cardiovascular Branch National Heart, Lung, and Blood Institute Division of Intramural Research Bethesda, MD United States; 4 Diabetes, Endocrinology and Obesity Branch National Institute of Diabetes and Digestive and Kidney Diseases Bethesda, MD United States

**Keywords:** psoriasis, calorie restriction, intermittent fasting, time-restricted eating, immunoregulation, immunometabolism

## Abstract

**Background:**

Psoriasis is a chronic inflammatory disease associated with multiple comorbidities, including metabolic syndrome and cardiovascular disease. Although specific dietary interventions, such as intermittent fasting and caloric restriction, have been shown to ameliorate inflammation and promote weight loss, the effect of these interventions independent of weight loss remains unclear. Time-restricted eating (TRE), a type of intermittent fasting, limits the daily eating window to a fixed number of hours. Recent studies suggest TRE may improve immune function in individuals with metabolic syndrome and cardiovascular risk factors. A crucial advantage of TRE over other investigated dietary restriction strategies is its reported high adherence rate, making it a more feasible intervention for long-term use. Therefore, exploring the effects of TRE on metabolic and immunological parameters in psoriasis is warranted.

**Objective:**

This study was designed to evaluate the effects of short-term, isocaloric TRE, independent of weight loss, on immune cell function and serum metabolite profiles of volunteers with mild-to-moderate psoriasis compared to healthy individuals.

**Methods:**

This case-control, prospective study was performed on 10 healthy male participants and 10 age-, BMI-, and sex-matched individuals with mild-to-moderate psoriasis. All individuals with psoriasis had stable disease and were being treated with topical therapies without any exposure to immunomodulatory biologics. This study was conducted at the National Institutes of Health Clinical Center. Immune profiles, glucose handling, energy expenditure, and participants’ weights were assessed at baseline and after 3 days of TRE following a daily 6-hour eating window and 18-hour fast.

**Results:**

The trial commenced in June 2021 and was completed in February 2023. A total of 20 participants were enrolled—10 with mild-to-moderate psoriasis and 10 age-, BMI-, and sex-matched healthy individuals. As of the time of manuscript submission, data processing is ongoing. Multiomic datasets, including gene expression, surface and intracellular protein levels, and metabolite profiles, are being generated from peripheral blood mononuclear cells, CD4^+^-enriched T-cells, and serum samples. The integrated bioinformatics analyses will be reported once the data analysis is completed.

**Conclusions:**

This clinical protocol was designed to characterize the effects of short term (3-day) TRE on psoriasis, independent of weight loss, by comparing immune cell regulatory responses between healthy individuals and those with psoriasis. More specifically, we aim to map the molecular pathways activated by TRE and assess how they affect immune cell composition, activation, and metabolism. Additionally, components of the metabolic response to isocaloric TRE are being explored. Insights into how dietary interventions impact metabolism and the immune system will enhance our understanding of the pathogenesis of psoriasis and may reveal new therapeutic avenues for managing this inflammatory condition.

**Trial Registration:**

ClinicalTrials.gov NCT04728165; https://clinicaltrials.gov/study/NCT04728165

## Introduction

Psoriasis is a chronic inflammatory skin disease that affects approximately 2%-3% of the US population. Beyond its skin and joint manifestations, psoriasis predisposes individuals to premature cardiovascular comorbidities, primarily due to immune activation and systemic, inflammation-induced atherosclerosis.[1] Skin-initiated immune activation through nonprofessional immune cells, including keratinocytes and fibroblasts, initiates and amplifies both innate and adaptive immune responses. These responses include the activation of monocytes and macrophages, neutrophils and their subsets (including low-density granulocytes), as well as effector T- cells (predominantly T_H_1 and T_H_17 subsets). These immune cells exacerbate local disease and propagate systemic sterile inflammation. The centrality of immune activation in psoriasis is reinforced by the therapeutic efficacy of targeted therapies, such as anti-TNF and anti-IL-17 treatments, which aim to modulate these immune responses [2,3].

The contribution of excess calorie consumption to the pathogenesis of psoriasis has been long debated. However, epidemiological and meta-analytic data support the notion that the incidence of obesity is higher in individuals with psoriasis and that the severity of psoriasis is positively correlated with an increased incidence of obesity [4]. The role of calorie excess in the pathophysiology of psoriasis is further strengthened by studies demonstrating the beneficial effects of calorie restriction on disease activity [5,6]. Taken together, these data suggest that calorie intake may play a significant role in the pathophysiology of psoriasis and that dietary restriction interventions may contribute to disease amelioration by modulating inflammatory pathways.

Various dietary interventions, including prolonged fasting [7,8], intermittent fasting, fasting mimetic dietary supplementation, and calorie restriction per se, have all been shown to confer anti-inflammatory effects through mechanisms that are not yet fully understood [9]. The underlying biological mechanisms are complex, as the duration of fasting may act as an independent mediator, distinct from the effects of calorie restriction on inflammation. Moreover, distinguishing cell-mediated anti-inflammatory effects from other aspects of dietary interventions, such as changes in glucose sensitivity or weight loss, remains challenging.

Previously, we showed that a single 24-hour fast is sufficient to blunt inflammation in monocytes and CD4^+^ T cells [7,8,10]. In contract, the direct assessment of inflammatory markers in time-restricted eating (TRE), which involved an 8-hour feeding window and 16-hour fast, showed modest effects on some markers (eg, reductions in circulating cytokine levels of TNFa and IL-1b, but not IL-6 and hs-CRP) [11]. In this context, we opted to test a more vigorous TRE protocol comprising a 6-hour eating window and 18-hour fast to assess the effects of this dietary intervention on immunoregulation.

To explore these mechanisms, we designed an acute exploratory study to examine the immune and metabolic responses to an isocaloric, short-term (3-day) TRE regimen consisting of a 6-hour eating window and an 18-hour fasting period in both healthy individuals and individuals with psoriasis. The primary outcome of this study was to phenotype circulating immune cells in both cohorts before and after TRE, focusing on the composition and activation status of peripheral blood mononuclear cells (PBMCs), T-cell subsets (specifically T_H_1 and T_H_17), and other immune mediators implicated in psoriasis pathogenesis. In parallel, the interplay of metabolic changes will be assessed by quantifying glucose control, weight changes, and energy expenditure.

We aimed to investigate the impact of short-term TRE in individuals with mild-to-moderate psoriasis compared with healthy individuals. We hypothesized that a 6-hour eating window followed by an 18-hour fast for 3 consecutive days would lead to changes in serum metabolites and immune cell composition, activation status, and cellular metabolism independent of glucose control and energy expenditure. Hence, this study aims to provide mechanistic evidence of how TRE ameliorates inflammation, offering insight into its benefits for psoriasis management and evaluating whether these effects are independent of weight loss. Furthermore, this initial limited study may serve as a proof-of-principle for future longitudinal investigations into the role of TRE in inflammatory conditions, including psoriasis and other related disorders.

## Methods

### Study Design

This study explored the immunometabolic features of a calorie restriction intervention by comparing the effect of TRE (6-hour eating window/18-hour fast) to a more conventional dietary regimen (12-hour eating window/12-hour fast). The conventional regimen was defined as the baseline for this study. Data were collected from individuals with mild-to-moderate psoriasis and from age-matched (within 10 years) and BMI-matched (within 5 kg/m^2^) healthy individuals. This is a small, acute TRE, case-control study where only male participants were enrolled to eliminate the confounding variable of menstrual cycle phases on low-grade inflammation and insulin sensitivity [12].

Participants were screened with a medical history and medicine review, physical exam, blood tests, skin examination, nutritional evaluation, and resting energy expenditure measurements. Study participants were admitted as inpatients to the Metabolic Clinical Research Unit at the National Institutes of Health (NIH) Clinical Center, where they stayed in individual rooms for 5 days. The first and final 23 hours were spent in metabolic chambers to measure baseline and TRE effects on energy expenditure.

### Study Intervention

The study was designed to explore the effects of TRE using a 6-hour eating window and an 18-hour fasting window. Participants followed a weight-maintaining balanced diet to meet 100% of their daily caloric needs throughout the inpatient visit. All foods and beverages were consumed in their rooms during designated hours of the day. On day 0, participants ate during a typical 12-hour window from 8 AM to 8 PM and then fasted from 8 PM to 8 AM the following day. From days 1-4, participants consumed all calories within a 6-hour eating window (8 AM to 2 PM), followed by an 18-hour fast. Meals were provided at 8 AM, 10:30 AM, and 1:30 PM, with participants given 30 minutes to consume each meal. Throughout the study, participants consumed a eucaloric diet consisting of 50% carbohydrate, 35% fat, and 15% protein. Each meal provided 33% of the participant’s estimated daily energy requirements. Energy needs for the study diet were calculated based on the resting energy expenditure measured at the screening visit and an activity factor of 60%. Participants had access to water throughout the study intervention.

Blood samples for immune and metabolic measurements were taken via intravenous catheter on day 1 and day 4. Participants wore a continuous glucose monitor (CGM) throughout the 5-day inpatient stay. Liquid mixed meal tests (MMTs) were conducted on day 1 (baseline) and day 4 (after 3 days of TRE). On MMT days, participants consumed a liquid meal for breakfast.

### Ethical Considerations

This study has been registered on Clinicaltrials.gov (NCT04728165). This study was approved by the National Institutes of Health Institutional Review Board (000149H). Informed consent was obtained both verbally as well as in writing prior to participation in the study. The data was deidentified by excluding the main identifying elements such as name, address, date of birth, phone number, email, medical record number, institutional identification number, and social security number. Participants received an identification code, according to the order of inclusion in the study. The participants were informed about the possibility of withdrawing at any time, without loss of their treatment at the institution. Additionally, participants were informed about the voluntary nature of their participation. Participants received financial compensation for their time and inconvenience of procedures due to no direct benefit for their inclusion in the study.

### Primary Objective

The primary objective of this study was to evaluate the response to TRE in individuals with psoriasis compared to age-, sex-, and BMI-matched healthy individuals. The primary outcome was the change in IL-17 secretion from activated CD4^+^ T cells at baseline and the end of the study in both groups.

### Secondary Objectives

The secondary objectives of this study were to compare the broader impact of TRE on immune cell regulatory pathways and immunometabolism. Additionally, we aimed to evaluate whether TRE modifies 24-hour glucose flux and glycemic excursions during meal tests. Energy expenditure was also compared at baseline and following 3 days of TRE. These measurements were taken over a 23-hour period in a metabolic chamber. Finally, the change in weight over the acute isocaloric study was determined.

### Recruitment and Eligibility

A total of 10 male participants aged 18 to 80 years with mild-to-moderate active psoriasis (measured using the psoriasis area and severity index score) were enrolled in the study. Ten age-matched (within 10 years) and BMI-matched (within 5 kg/m^2^) healthy individuals were also enrolled as the control group. The inclusion and exclusion criteria are outlined in Textbox 1.

**Inclusion and exclusion eligibility criteria for study participation.**

**Inclusion criteria:**

- Men aged 18 to 80 years with mild-to-moderate active psoriasis, as determined using the psoriasis area and severity index score.
- Age-matched (within 10 years) and BMI-matched (within 5 kg/m^2^) healthy men for inclusion in the control group.
- Individuals able to provide informed consent.
- Individuals willing and able to participate in the study procedures.

**Exclusion criteria:**

- A psoriasis area and severity index score above 12.
- Individuals who received treatment with systemic biologic immune modifying agents within the last 2 months.
- Individuals currently receiving treatment for allergies or other inflammatory diseases.
- Individuals who have taken vitamin B or tryptophan supplementation within 2 weeks of participation.
- Individuals unwilling or unable to provide informed consent.
- Individuals with a known history of type 1 or 2 diabetes mellitus or other metabolic conditions that would interfere with study parameters, including chronic kidney disease, chronic liver disease, or history of hypoglycemia.
- Individuals using medications that would interfere with the study parameters, including antihyperglycemic medications, systemic steroids, adrenergic-stimulating agents, or other medications known to affect sleep, circadian rhythms, or metabolism.
- Individuals with caffeine consumption in excess of three 8 oz cups per day.
- Individuals exposed to factors affecting circadian rhythm, such as overnight shift work, irregular sleep or eating schedules, or regularly fasting for more than 15 hours per day.
- Individuals who have regularly used tobacco products within the last 3 months.
- Individuals consuming more than 3 servings of alcohol per day.
- Individuals engagement in competitive sports training.
- Individuals with moderate to severe claustrophobia.
- Individuals with unstable weight (ie, with more than a 5% change in body weight in the last 3 months).
- Individuals with food allergies or intolerances or dietary patterns that would prohibit consumption of the study diet or mixed meal test.

### Study Interventions

The participants were required to fast for 12 hours prior to the screening visit, during which fasting energy expenditure was measured using the ventilated hood technique. This assessment was used to calculate isocaloric diets for each individual, which they followed during the conventional and TRE regimens.

On admission to the metabolic unit on day 0, a CGM was placed following an initial breakfast meal. Total energy expenditure was determined using indirect calorimetry during a 23-hour stay in the respiratory chamber. Participants entered the respiratory chamber after breakfast on day 0 and day 4. At 8 AM on day 1, blood samples were drawn for the immune parameter studies. Following this, participants underwent a MMT at breakfast after completing the conventional regimen (day 1) to measure metabolic hormones and serum metabolites.

**Figure 1:**
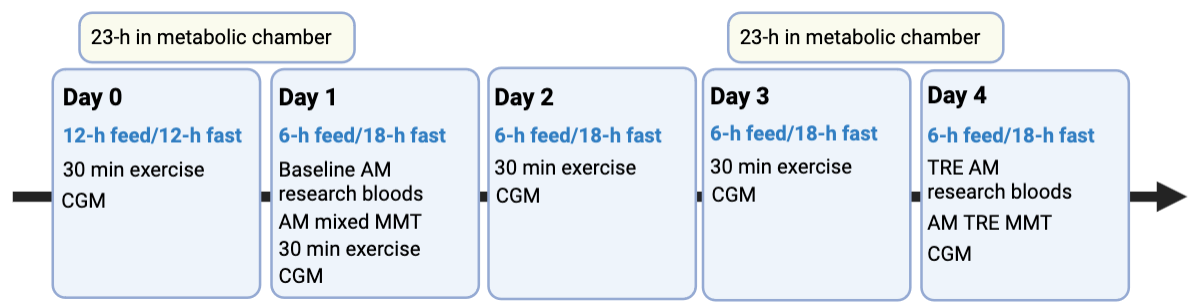
Schematic of the clinical study. CGM: continuous glucose monitoring; MMT: mixed meal test; TRE: time-restricted eating.

Participants then initiated the isocaloric TRE regimen for 3 days. On day 3, after breakfast, they entered the respiratory chamber again for a 23-hour analysis to assess the potential metabolic effects of the TRE protocol. On the following morning (day 4), blood samples were drawn at 8 AM for immune parameter studies after completing the third 18-hour fast. While still in the respiratory chamber, participants underwent a final MMT to assess the metabolic features of TRE. The study was completed once participants exited the chamber. The schematic of the study is shown in Figure 1.

### Summary of Tests and Calculations

#### Anthropometrics

Participants’ weights and heights were measured on day 0. Daily weight measurements were subsequently obtained from day 1 to day 4.

#### Metabolic Cart

Resting energy exposure was measured to assess basal energy requirements for designing the eucaloric diets for each study participant. Measurements were performed in the morning after a 12-hour fast using indirect calorimetry with the ventilated hood technique with a metabolic cart (Parvomedics, Murray, UT) [13].

#### Mixed Meal Test

After a 12-hour (day 0) and 16-hour (day 4) overnight fast, 1 intravenous catheter was placed in each arm for blood draws upon arrival in the morning. A liquid meal (Boost Plus) was provided to meet 33% of the estimated daily calorie requirements. Blood samples were obtained after 0, 10, 20, 30, 40, 50, 60, 90, and 120 minutes to measure plasma glucose, serum insulin and C-peptide, and triglyceride concentrations [14].

#### Respiratory Chamber

Total body energy expenditure was assessed in an in-room metabolic respiratory chamber over 23 hours beginning on day 0 and day 3 [15]. In this isolation chamber, resting and sleeping energy expenditure, the thermic effect of food [16], and the energy cost of physical activity can be assessed [17]. Blood samples were obtained through an isolette system, allowing direct nursing contact with the participant. Telemetry and a nurse call were available to ensure participant safety. A 24-hour urine specimen was collected while in the chamber to measure changes in urea and nitrogen levels. A random urine creatine sample was also collected.

Respiratory chamber measurement periods were approximately23 hours, and the data were extrapolated to represent 24-hour periods by assuming the mean of the measured periods accurately represented the full 24-hour period. The formula used to calculate energy expenditure was as follows: energy expenditure (kcal) = 3.88 × VO_2_ – 0.32 L/g × Kexcr (g) + 1.08 × VCO_2_ - 1.57 × N. VO_2_ and VCO_2_ are the volumes of oxygen consumed and carbon dioxide produced, respectively, in liters. Kexcr refers to ketones excreted in grams. N refers to the 24-hour urinary nitrogen excretion in grams.

Sleeping energy expenditure was determined as the lowest energy expenditure over a continuous 180-minute period between midnight and 6 AM.

The ratio of VCO_2_ to VO_2_ is termed the respiratory exchange ratio and reflects the relative oxidation of macronutrients (carbohydrates, fats, and protein). Changes in the respiratory exchange ratio from waking hours to the fasted sleeping period were used to quantify metabolic flexibility during each feeding schedule [14].

#### Continuous Glucose Monitoring

Participants wore FDA-approved CGMs during their inpatient admission. The devices recorded real-time serum glucose levels approximately every 5 minutes. The system consisted of a small sensor, transmitter, and handheld receiver (roughly the size of a pager). The sensor was inserted with a small introducer needle that was retracted after subcutaneously inserting the sensor. The transmitter, attached to the sensor, sent the measured glucose readings to the receiver. The data stored in the receiver was uploaded to a server for future analyses. To compare the change in 24-hour glycemic profiles, glucose data from day 0 at noon will be compared to data from day 4. A CGM R package was used to calculate active wear time, wear days, percent wear days, and glycemic metrics, including average blood glucose, time in range, SD, coefficient of variation, and mean amplitude glucose excursion [18,19].

#### Metabolic Analyses

Fasting concentrations of glucose, insulin, C-peptide, and triglycerides were measured in serum using the Roche Cobas 6000 analyzer (Roche Diagnostics). Hemoglobin A1c was measured using the HPLC D10 instrument (Bio-Rad).

#### PBMC Multiomic Analysis

TRE-mediated changes in immune cells will be assessed using high-throughput single-cell modalities. Transcriptomic and immunophenotype changes will be quantified using single cell RNA-sequencing (scRNA-seq) combined with cellular indexing of transcriptomes and epitopes sequencing (CITE-seq). Changes in cellular metabolism will be assessed using cytometry by time-of-flight (CyTOF) to quantify levels of metabolic enzymes and regulatory proteins [20]. This approach has been successfully performed in similarly modest-sized human studies [21]. The integration of these datasets will enable an unprecedented, granular analysis of immune cells before and after TRE in both healthy individuals and patients with psoriasis. Downstream analyses will be performed to generate hypotheses that may be tested through ex vivo studies [8,22] or inform the design of future larger and longer-term prospective studies.

### Outcome Measures

The primary outcome was quantification of IL-17 secretion from T cell receptor–activated CD4+ T cells isolated from samples collected on day 1 and day 4. The delta (change) in IL-17 secretion was compared using a 2-tailed paired *t* test. IL-17 secretion in response to TRE was compared between the 2 cohorts using a 2-tailed, 2-sample *t* test in each study group.

The collected samples will also allow hypothesis generation to understand the molecular mechanisms underlying changes in IL-17 secretion. To this end, PBMC transcriptomic and protein-level changes will be quantified using high-throughput multimodal platforms, including scRNA-seq, CITE-seq, and CyTOF. A broad range of downstream analyses will be performed to uncover immunophenotypes, cellular states, enriched signaling pathways, and intercellular communication. More specifically, clustering and cell type annotation will allow the identification of distinct cell populations based on gene and protein expression profiles. Differential gene expression analysis will elucidate significant expression changes pre- and post-TRE. Gene set enrichment analysis will be used to evaluate the enrichment of specific pathways or biological processes. Cell-to-cell communication inference tools will identify putative ligand-receptor interactions driving immune responses. Moreover, multimodal integration will permit the cross-validation of cell types using both gene and protein expression data and link transcriptional programs to functional immune phenotypes.

We will implement statistical correction tools across all data types to address the risk of false discoveries due to multiple hypothesis testing. This will include false discovery rate adjustment using the Benjamini-Hochberg method.

For phenotypic data, including glucose levels and metabolic rates, comparisons within groups were conducted using paired 2-tailed *t* tests, and between-group comparisons were performed using unpaired 2-tailed *t* tests. A *P* value of <.05 was considered statistically significant.

### Sample Size Calculations and Statistical Analyses

There are no published data on TRE in either healthy individuals or individuals with psoriasis on which to base calculations for the number of participants required to uncover distinct effects on IL-17 cytokine release. Hence, to justify our sample size and facilitate power calculations for 10 participants per group, we referred to data from a previous fasting study in which isolated CD4^+^ T cells were analyzed in 13 healthy individuals at the NIH Clinical Center (ClinicalTrials.gov NCT02719899). In that study, the mean and SD for the paired refed-fasting differences in IL-17 release were 187.1 pg/ml and 398.79 pg/ml, respectively [8].

For the primary objective, our computations for change from baseline IL-17 secretion in patients with psoriasis assumed the SD from the aforementioned fasting study. Using a 2-tailed paired *t* test with α=.05 and n=10, we estimated 80% power to detect a clinically significant change of 396.36 pg/ml or greater. To compare the change in IL-17 secretion between healthy participants and patients with psoriasis, we assumed a 187.1 pg/ml change from baseline in controls (the value observed in the fasting study). With a 2-tailed paired *t* test with α=.05 and n=10 for the 1:1 matched participants, the study would have 85% power to detect a 209.26 pg/ml (396.36 pg/ml – 187.1 pg/ml) or greater difference between the psoriasis and control groups in IL-17 secretion from baseline. For these computations, we assumed a SD of 199.4 pg/ml for the paired differences in IL-17 secretion from baseline between matched patients with psoriasis and healthy participants (half the SD for the controls in the fasting study).

Therefore, we requested approval to enroll up to 15 healthy individuals and 15 individuals with mild-to-moderate psoriasis for this initial study, with the goal of having at least 10 participants complete the study in each group. The effect of TRE will be assessed within each group and the response to TRE will be assessed between groups.

All other analyses will be exploratory, and the analytical approaches are briefly described in the Outcome Measures section above.

### Dissemination of Project Findings

The findings of this study will be disseminated through various channels to reach a diverse audience. To engage the dermatology clinical community, we aim to share our results at international and national congresses focusing on dermatology and psoriasis. Additionally, our findings will be disseminated to the scientific communities focused on immunology and metabolism by publishing our findings in peer-reviewed journals germane to those fields. We seek to engage the patient community through potential collaborations with the National Psoriasis Foundation and via a laypeople summary of the findings. Finally, we want to reach the general public by communicating the key results through social media platforms.

## Results

Patients were enrolled in the study between June 2021 and February 2023. PBMC scRNA-seq, CITE-seq, and CyTOF were performed in 2024. Data analysis is currently being performed concurrently with flow cytometry and CD4^+^ T cell metabolomics. We aim to publish and share our findings by 2026.

## Discussion

In psoriasis, the major circulating immune cell lineages operational in the disease pathophysiology include neutrophils, low-density granulocytes, monocytes, and CD4^+^ T cells [23-25]. This study was designed to explore the gene regulatory and metabolic pathways altered in most of these cells, except for neutrophils, which are excluded in the polymorphonuclear cell fraction isolation protocol. Including a control group will allow us to evaluate whether the immune effects of TRE are more pronounced in an inflammatory disease cohort compared to individuals without baseline inflammation.

The primary outcome of this study, aimed at determining T_H_17 immune responsiveness through IL-17 secretion, was performed in negatively selected T cell receptor–activated CD4^+^ T cells. As the samples were paired (baseline and post-TRE), these CD4^+^ T cells were also analyzed for changes in intracellular and serum steady-state metabolite levels. This analysis will help characterize whether TRE evokes systemic metabolite changes that influence CD4^+^ T cell signaling and provide insights into how TRE modulates intracellular metabolic pathways.

From an unbiased discovery perspective, data from high-throughput multimodal technologies, including scRNA-seq, CITE-seq, CyTOF, and flow cytometry, will be analyzed and integrated [26] to explore the effects of TRE in both healthy individuals and individuals with psoriasis. These analyses will offer crucial hypothesis-generating insights into how TRE exerts anti-inflammatory effects across different immune cell populations in PBMCs.

Finally, this initial study was designed to evaluate whether the effects of weight loss or glucose sensitivity can be dissociated from the direct immunomodulatory effects of TRE. The robustness of the eucaloric study design will be determined by changes in weight, continuous glucose monitoring, and energy expenditure, as measured by indirect calorimetry.

The limitations of this study arise in part from its small sample sizes and its short duration (3 days of TRE). This design is intended to generate hypotheses about immunoregulation independent of caloric load that can be further explored in more targeted and larger studies. Another limitation of the small study size was the restriction of this protocol to men to alleviate adding the variable of hormonal changes during the menstrual cycle. This is obviously a significant constraint when considering the generalizability of findings to women, and this will need to be addressed in a larger, longer-term follow up study.

## Abbreviations

CGM: continuous glucose monitor
CITE-seq: cellular indexing of transcriptomes and epitopes sequencing
CyTOF: cytometry by time-of-flight
MMT: mixed meal test
NIH: National Institutes of Health
PBMC: peripheral blood mononuclear cell
scRNA-seq: single cell RNA-sequencing
